# Patient-derived organoids of pancreatic ductal adenocarcinoma for subtype determination and clinical outcome prediction

**DOI:** 10.1007/s00535-024-02103-0

**Published:** 2024-04-29

**Authors:** Kazuhide Matsumoto, Nao Fujimori, Kazuya Ichihara, Ayumu Takeno, Masatoshi Murakami, Akihisa Ohno, Shotaro Kakehashi, Katsuhito Teramatsu, Keijiro Ueda, Kohei Nakata, Osamu Sugahara, Takeo Yamamoto, Akinobu Matsumoto, Keiichi I. Nakayama, Yoshinao Oda, Masafumi Nakamura, Yoshihiro Ogawa

**Affiliations:** 1 Department of Medicine and Bioregulatory Science, Graduate School of Medical Sciences, Kyushu University, 3-1-1 Maidashi, Higashi-ku, Fukuoka, 812-8582 Japan; 2 Department of Molecular and Cellular Biology, Medical Institute of Bioregulation, Kyushu University, Fukuoka, Japan; 3 Department of Surgery and Oncology, Graduate School of Medical Sciences, Kyushu University, Fukuoka, Japan; 4 Department of Anatomic Pathology, Graduate School of Medical Sciences, Kyushu University, Fukuoka, Japan

**Keywords:** Pancreatic ductal adenocarcinoma, Patient-derived organoids, Precision medicine, Subtype classification, Turnaround time

## Abstract

**Background:**

Recently, two molecular subtypes of pancreatic ductal adenocarcinoma (PDAC) have been proposed: the “Classical” and “Basal-like” subtypes, with the former showing better clinical outcomes than the latter. However, the “molecular” classification has not been applied in real-world clinical practice. This study aimed to establish patient-derived organoids (PDOs) for PDAC and evaluate their application in subtype classification and clinical outcome prediction.

**Methods:**

We utilized tumor samples acquired through endoscopic ultrasound-guided fine-needle biopsy and established a PDO library for subsequent use in morphological assessments, RNA-seq analyses, and in vitro drug response assays. We also conducted a prospective clinical study to evaluate whether analysis using PDOs can predict treatment response and prognosis.

**Results:**

PDOs of PDAC were established at a high efficiency (> 70%) with at least 100,000 live cells. Morphologically, PDOs were classified as gland-like structures (GL type) and densely proliferating inside (DP type) less than 2 weeks after tissue sampling. RNA-seq analysis revealed that the “morphological” subtype (GL vs. DP) corresponded to the “molecular” subtype (“Classical” vs. “Basal-like”). The “morphological” classification predicted the clinical treatment response and prognosis; the median overall survival of patients with GL type was significantly longer than that with DP type (*P* < 0.005). The GL type showed a better response to gemcitabine than the DP type in vitro, whereas the drug response of the DP type was improved by the combination of ERK inhibitor and chloroquine.

**Conclusions:**

PDAC PDOs help in subtype determination and clinical outcome prediction, thereby facilitating the bench-to-bedside precision medicine for PDAC.

**Supplementary Information:**

The online version contains supplementary material available at 10.1007/s00535-024-02103-0.

## Introduction

Pancreatic ductal adenocarcinoma (PDAC) is one of the most lethal cancers, with a 5-year survival rate of approximately 10%. It is the fourth leading cause of cancer-related deaths worldwide [1], and its incidence and mortality rates are increasing. More than 80% of patients are diagnosed with unresectable disease at the time of diagnosis because of the difficulty in early detection [2]. Currently, the selection of standard chemotherapies for metastatic PDAC, such as FOLFIRINOX (FFX) [3] and gemcitabine (GEM) plus nab-paclitaxel (GnP) [4], is based on the patient performance status (PS) and comorbidities. Therefore, treatment response varies among patients with PDAC, and chemotherapy often results in unfavorable clinical outcomes. Accordingly, the poor survival rate has improved only modestly over the past decades [2], and the overall survival time (OS) remains unsatisfactory, with a median of 8.5–11.1 months [3, 4]. Cellular heterogeneity in PDAC causes resistance to treatment [5]. Thus, early detection methods and effective therapeutic strategies are required to improve the outcomes of patients with PDAC [6, 7].

Recent advances in cancer genome medicine have resulted in the development of new treatment strategies for various cancer types, such as lung and colorectal cancers [8, 9]. However, in PDAC, the effects of cancer genome medicine are limited and insufficient. Some new therapeutic agents for PDAC are available: for example, pembrolizumab for high microsatellite instability and high tumor mutation burden [10, 11], and olaparib for germline *BRCA* mutations [12]. However, very few targeted mutations have been identified, and the turnaround time from sample collection to genotype diagnosis and chemotherapy selection is too long to provide patients with precision medicine at the bedside. Indeed, only 10% of patients are treated with pharmacological agents based on genomic testing outcomes [13].

Recent advances in molecular genetics have led to the proposal of two molecularly defined subtypes of PDAC [14–18]: the “Classical” and “Basal-like” or ‘‘Squamous’’ subtypes, with the former showing better treatment response and prognosis than the latter. In several studies, patient-derived organoids (PDOs) have been used to elucidate the complexity of PDAC because they can maintain in vivo tumor cell diversity. Indeed, single-cell RNA-seq (scRNA-seq) analysis of PDOs revealed cellular heterogeneity in both subtypes [19, 20]. However, it is also noteworthy that subtypes of PDAC often shift from “Classical” to “Basal-like” as the disease progresses [21]. PDOs could provide a breakthrough in elucidating the mechanisms of progression to malignancy [22] and in pharmacotyping [23, 24].

Despite the marked heterogeneity of PDAC, subtype classification has not yet been applied in real-world clinical practice. Although some candidate molecules such as GATA6 and CK5 have been considered as surrogates for “Classical” and “Basal-like” subtypes, respectively, for research purposes [25, 26], it is difficult to determine the subtypes to be used for treatment selection in standard medicine. If the subtypes can be easily determined, the treatment option for PDAC will be optimized as “precision medicine.” For instance, in Canada, a prospective clinical trial using GATA6 as a “Classical” biomarker is currently underway (NeoPancONE) [27]. In contrast, no effective therapeutic targets for “Basal-like” have been identified. Some clinical trials focusing on new therapeutic agents, such as an autophagy inhibitor [28], are in progress; however, they have not been applied for practical use, and hurdles for clinical application remain.

In this study, we established PDOs for PDAC with high efficiency, which aided in subtype classification and clinical outcome prediction. The PDOs provide a unique experimental system with which to assess new therapeutic targets, thereby facilitating the “bench-to-bedside type” precision medicine for PDAC.

## Methods

### Patients

We performed a prospective study on patients diagnosed with pancreatic neoplasms at Kyushu University Hospital between April 2020 and March 2023. Informed consent was obtained from all patients. In this study, we focused on PDAC. Clinical data of patients with PDAC, including information about age, sex, pathological diagnosis, presence of metastases, Union for International Cancer Control (UICC) staging, treatment procedures, and prognosis, were obtained from electronic medical records. This study was approved by the Ethics Committee of Kyushu University (approval number: 22121-00) and was conducted according to the Ethical Guidelines for Human Genome/Gene Research enacted by the Japanese Government and the Helsinki Declaration.

### Samples

Tumor samples were obtained by endoscopic ultrasound-guided fine needle biopsy (EUS-FNB), liver biopsy, surgical resection, abdominal paracentesis, and endoscopic retrograde cholangiopancreatography (ERCP). Single-pass EUS-FNB was performed using a 19G or 22G needle (Acquire™; Boston Scientific, Marlborough, MA, USA; TopGain; MediGlobe, Tempe, AZ, USA) as previously reported [29]. Percutaneous biopsy of liver metastases was performed using a 16G or 21G lancet needle. The other samples obtained were as follows: 30–50 mm^3^ of surgical resection tissue; 100–200 mL of ascites via percutaneous abdominal paracentesis; and 5–10 mL of pancreatic juice via ERCP.

### PDOs

**Fig. 1:**
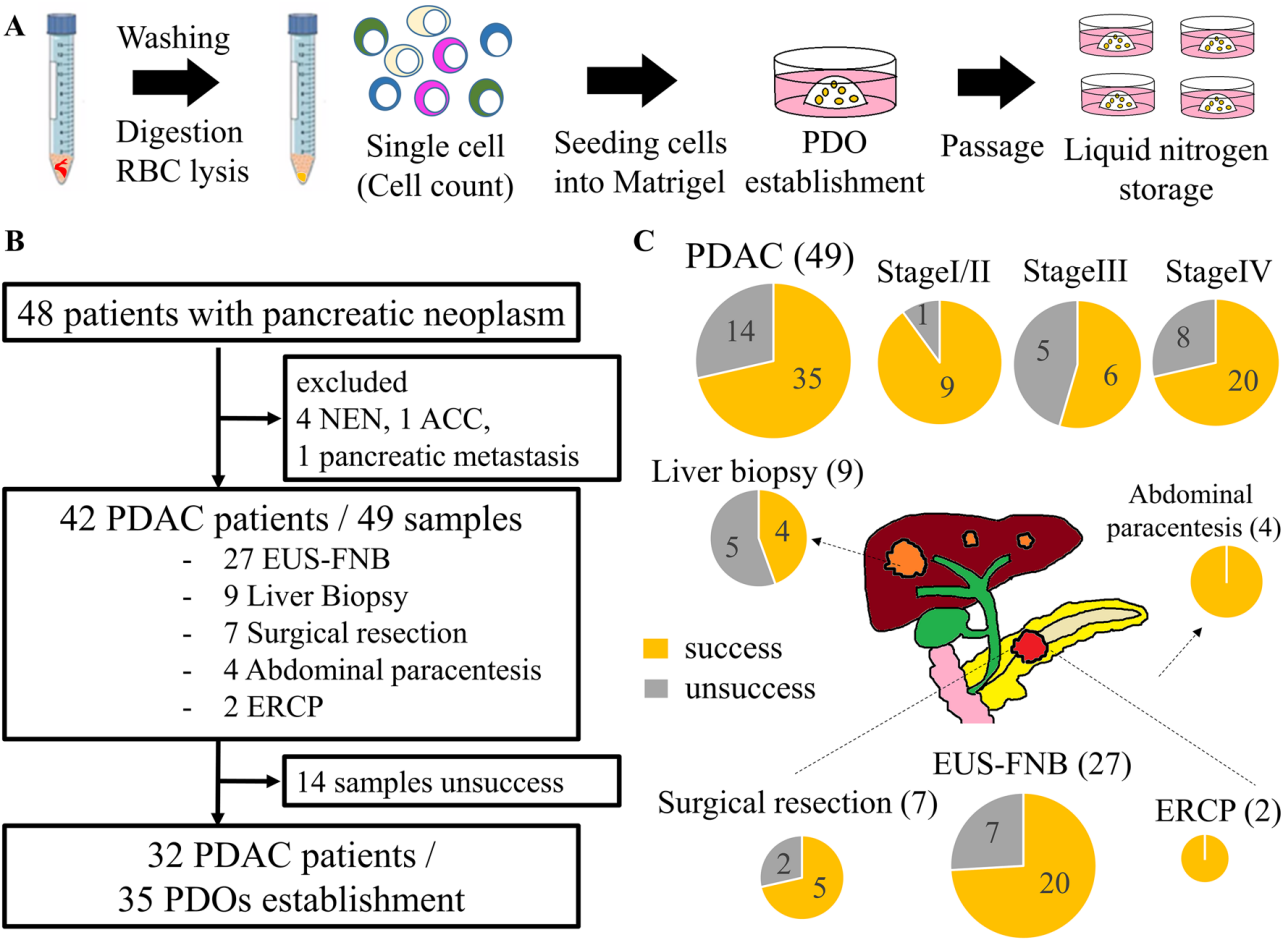
**A** Illustration of the process involved in PDO establishment. Tumor samples underwent digestion into single cells, followed by embedding in Matrigel, and culture in complete medium to generate PDOs (patient-derived organoids). **B** Flowchart of this study. *NEN* neuroendocrine neoplasm, *ACC* acinar cell carcinoma, *PDAC* pancreatic ductal adenocarcinoma, *EUS-FNB* endoscopic ultrasound-guided fine-needle biopsy, *ERCP* endoscopic retrograde cholangiopancreatography (**C**), Success rates of PDO establishment using different sampling methods, highlighting the utility of EUS-FNB in PDO generation

PDOs were established according to previous reports, with slight modifications [30, 31]. Solid PDAC samples obtained via EUS-FNB, surgical resection, and liver biopsy were washed with ice-cold phosphate-buffered saline (PBS). The surgically resected tissues were minced into approximately 1 mm^3^ fragments before digestion. Samples were digested into single cells using Liberase TH (20 min) and TrypLE Express (10 min) at 37 °C in a water bath (with pipetting every 5 min). After washing with basal medium (Supplementary Table S1), the red blood cells (RBCs) were hemolyzed in RBC lysis buffer for 10 min. Liquid samples, such as ascites and pancreatic juice obtained via ERCP, were washed with basal medium. The dissociated cells were embedded in Matrigel (356,231; Corning, NY, USA) and cultured in complete medium (Supplementary Table S1) in a 24-well plate (353,504; Corning) in a 37 °C incubator with 5% CO_2_ (Fig. 1A).

The culture medium was changed every 3–4 d. For passaging, the PDOs were collected and dissociated by digestion with TrypLE Express for 3–5 min. The cells dissociated from the PDOs were replated with fresh Matrigel and cultured in a complete medium. PDOs were cultured without EGF after passaging to enrich the KRAS mutations, as reported previously [22]. Successful establishment of PDOs was defined as success in passage five times. Images of the PDOs were acquired via fluorescence microscopy (BZ-X710; Keyence, Osaka, Japan), and analyzed using the ImageJ software (National Institutes of Health, MD, USA). The PDOs were assigned in the order of their establishment.

### Total RNA extraction and quantitative RT-PCR (qRT-PCR)

Total RNA was extracted from the frozen samples of surgically resected tumors and the cultured PDOs using the ISOGEN reagent (NIPPON GENE, Tokyo, Japan) according to the manufacturer’s instructions. qRT-PCR was performed using the CFX Connect Real-Time System (Bio-Rad Laboratories, Hercules, CA, USA). The double-stranded DNA-specific dye SYBR Green I was incorporated into the PCR buffer provided in TB Green Premix EX Taq II (Takara Bio Inc., Shiga, Japan) to enable quantitative detection of the PCR product. Transcript levels were determined using the ΔΔCt method and normalized to that of *ACTB* mRNA. The primer sequences are listed in Supplementary Table S2.

### RNA-seq and data analysis

The NEBNext rRNA Depletion Kit (New England Biolabs, Ipswich, MA, USA) was used to deplete ribosomal RNA from total RNA (500 ng), and the RNA was converted to an Illumina sequencing library using the NEBNext Ultra RNA Library Prep Kit for Illumina (New England Biolabs). The library was validated using a Bioanalyzer (Agilent Technologies, Santa Clara, CA, USA) to determine the size distribution and concentration, and sequenced on the NextSeq 500 (Illumina, San Diego, CA, USA) with the paired-end 36-base read option. Reads were mapped to the human reference genome hg19 using STAR version 2.7.3a and quantified using featureCounts version 1.6.4. Values were normalized as reads per kilobase of exons per million mapped reads. We integrated RNA-seq data using ComBat-seq to remove batch effects [32]. Gene ontology (GO) analysis was performed using Database for Annotation, Visualization, and Integrated Discovery software 2021. Gene set enrichment analysis (GSEA) was performed using GSEApy version 0.9.9. The hallmark and C6 (oncogenic signature) gene sets were downloaded from the Molecular Signatures Database (version 2022.1.Hs: http://www.broadinstitute.org/gsea/index.jsp). Squamous PDAC and Progenitor PDAC identity signatures [33], and Basal-like and Classical signatures [15] have been previously described.

### Analogy of genomic mutations

Genomic mutations such as SNPs and indels were inferred using the RNA-seq data. Somatic variant calling was performed as described previously [34]. Duplicate reads were flagged using Mark-Duplicates (Picard, version 2.27.4) and split into exons using SplitNCigarReads (GATK, version 4.2.6.1). Variant calling was performed using the Mutect2 (GATK, version 4.2.6.1).

### Immunohistochemistry (IHC) and immunofluorescence (IF) staining

Paraffin-embedded blocks of PDAC tissues were cut into 4 µm-thick sections and subjected to standard hematoxylin and eosin (H&E) staining and immunostaining. PDOs were isolated from Matrigel using Cell Recovery Solution (354,253; Corning) according to the manufacturer’s instructions. For IHC staining, sections were deparaffinized and rehydrated. Heat-mediated antigen retrieval was performed using sodium citrate buffer (pH6) for GATA6 and Tris/EDTA buffer (pH 9) for CK5. The sections were treated with 3% H_2_O_2_ in methanol to block endogenous peroxidase activity and then blocked with Blocking One Histo (Nacali Tesque, Kyoto, Japan). They were then washed with PBS/0.1% Triton X-100 and incubated with primary antibodies against GATA6 (AF1700; R&D SYSTEMS, Minneapolis, MN, USA; 10 μg/mL) and CK5 (GTX113219; GeneTex, Irvine, CA, USA; 1:1000) for 1 h at room temperature. Subsequently, the sections were incubated with the appropriate secondary antibody: horseradish peroxidase (HRP)-conjugated anti-goat IgG antibody (ab6741; Abcam, Cambridge, UK; 1:1000), anti-rabbit IgG (ab6721; Abcam; 1:1000), Alexa Fluor 488 anti-rabbit (ab150077; Abcam; 1:500), and 555 anti-goat (ab150130; Abcam; 1:500). Thereafter, 3,3'-diaminobenzidine substrate chromogen solution (11,209-1A; Kanto Kagaku, Tokyo, Japan) was applied, followed by counterstaining with hematoxylin. For IF staining, the nuclei were counterstained with 4',6-diamidino-2-phenylindole (Dojindo, Kumamoto, Japan).

### Drug response assay

The PDOs were dissociated into single cells, and a 1000 live cells were seeded in a 96-well plate (B&W IsoPlate-96 TC; PerkinElmer, Kanagawa, Japan) containing Matrigel and complete medium. Therapeutic drugs, namely GEM (073-06631; FUJIFILM WAKO, Osaka, Japan; ranging from 1.0 × 10^−10^ to 1.0 × 10^−5^ mol/L), ulixertinib (BVD-523, HY-15816; MedChemExpress, NJ, USA; 1.0 × 10^−6^ mol/L), and chloroquine (HY-17589A; MedChemExpress, 5.0 × 10^−6^ mol/L) were added 24 h after plating and tested in triplicate. After 5 d, cell viability was quantified using the CellTiter-Glo 2.0 Kit following the manufacturer’s instructions (Promega, Madison WI, USA).

### Statistical analysis

All statistical analyses were performed using Microsoft Excel, JMP Pro statistical software (ver. 16; SAS Institute Inc., Cary, NC, USA), and GraphPad Prism 9 software (Graphpad Software, Inc., Boston, MA, USA). Comparisons between two groups were assessed using Welch’s *t* test for continuous variables (normal distribution), Wilcoxon rank-sum test for continuous variables (non-normal distribution), or Fisher’s exact test for categorical data. Survival curves were analyzed using the Kaplan–Meier method with the log-rank test. Statistical significance was set at *P* < 0.05.

## Results

### Patient characteristics and PDO establishment

A total of 48 patients with suspected pancreatic cancer were enrolled, of whom 42 (49 samples) were diagnosed with PDAC via histopathologic assessment, and 35 PDOs were established. The median age of the patients was 67 years, the male/female ratio was 18/14, and Stage I, II/III/IV was 9/6/17 (UICC 7th edition). Figure 1B shows the flowchart for the enrollment of patients in this study.

In this study, the overall success rate of PDO establishment was 71%. Meanwhile, the success rates of EUS-FNB, liver biopsy, and surgical resection were 74%, 44%, and 71%, respectively (Fig. 1C). Despite the limited number of cases, we established PDOs from the ascites and pancreatic juice obtained via ERCP. Successful establishment of PDOs depended on the number of live cells obtained after digestion, but not on the tumor stage and size (Table 1). Indeed, the establishment efficiency was significantly higher in samples with 100,000 or more live cells than in those with fewer than 100,000 (84% vs. 38%, *P* < 0.01). We also obtained more live cells using EUS-FNB than using liver biopsy (*P* < 0.01, Supplementary Fig. S1). We achieved a success rate of > 70% with a single puncture using a 22 G Franseen needle.

**Table 1:** Characteristics of patients enrolled in this study (excluding ascites cases)

	Samples (*n* = 45)		*P*
	Success (*n* = 31)	Failure (*n* = 14)	
Gender, male/female	19/12	7/7	0.528
Age (median)	64 (46- 81)	67 (53- 84)	0.531
Stage (UICC 7th)			0.137
I/II	9	1	
III/IV	22	13	
CA19-9 (median, U/mL)	246 (0–5310)	340 (0–28,669)	0.3639
Sampling method			0.2871
EUS-FNB	20	7	
Liver biopsy	4	5	
Surgical resection	5	2	
ERCP	2	0	
Tumor size (median, mm)	30 (5–51)	25 (12–45)	0.167
Live cell (median, × 10^4^)	39.4 (1.5–820.4)	8.9 (0–157.5)	0.0105*

### “Morphological” classification of PDOs

**Fig. 2:**
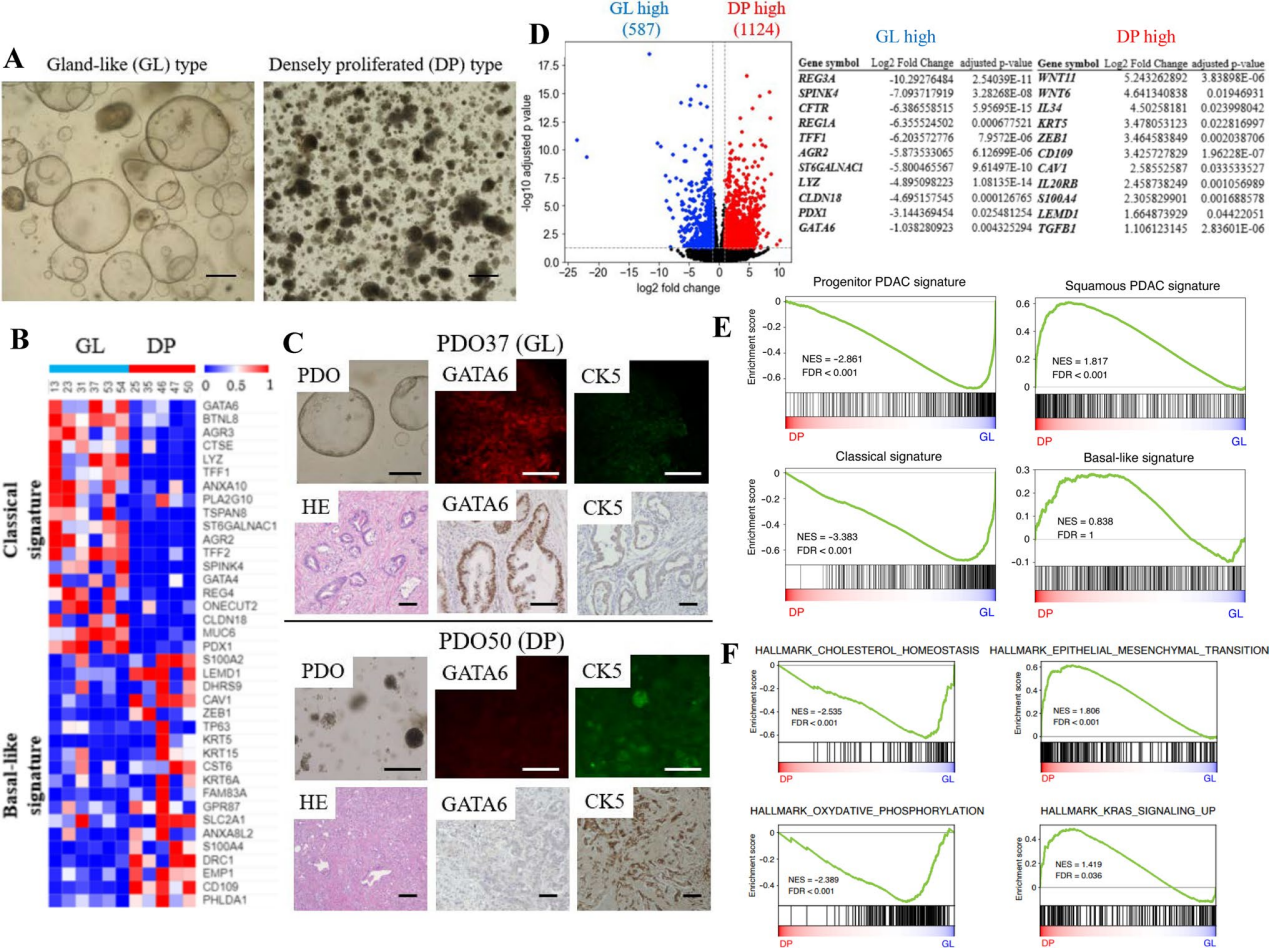
**A** “Morphological” classification of PDOs into GL and DP subtypes. Scale bar, 100 µm. **B** Heatmap of gene expression levels according to “Classical” and “Basal-like” signatures in PDOs. The bar indicates “morphological” classification. **C** PDO37, GL type (top); PDO50, DP type (bottom). Representative PDO images and IF staining of GATA6 (“Classical” marker) and CK5 (“Basal-like” marker) (above). HE and IHC staining of the resected tumors from which the organoids were established (below). *HE* hematoxylin and eosin. Scale bar, 100 µm. **D**–**F** RNA-seq analysis of the same PDOs as in 2B. The total number of samples was 11. **D** Volcano plot of DEGs for GL vs. DP (left). Major genes enriched in GL and DP are listed (right). **E** Enrichment of Progenitor PDAC signature (top left), Classical signature (bottom left), Squamous PDAC signature (top right), and Basal-like signature (bottom right) by GSEA of genes more highly expressed in GL and DP, respectively. NES; normalized enrichment score, FDR; false discovery rate *q* value. **F** Hallmark pathways significantly enriched among DEGs in GL and DP according to GSEA. Gene sets with high NES are shown

We performed a morphological evaluation of PDOs via image analysis and found two distinct subtypes: a gland-like structure (GL type) and a densely proliferating structure (DP type; Fig. 2A). After aligning the background brightness, contrast, and color tone, regions of interest (ROIs) were set for each PDO, and the means were compared. GL was defined as 100 or more, and DP was defined as less than 100. The “morphologic” subtypes were determined less than 2 weeks after tissue sampling. No significant differences in the frequency of genetic mutations in PDAC, *KRAS*, *CDKN2A*, *TP53*, *SMAD4*, *BRCA1/2*, *ARID1A*, and *KDM6A* were observed between the two subtypes (Supplementary Fig. S2A–C). Meanwhile, in RNA-seq analysis, the “morphological” classification corresponded to the “molecular” subtype; GL and DP types roughly corresponded to “Classical” and “Basal-like” subtypes, respectively (Fig. 2B). Details of the patient characteristics analyzed using RNA-seq are listed in Supplementary Table S3. PDO37, which was of the GL type, was a well to moderately differentiated adenocarcinoma by postoperative pathology, while PDO50, which was of the DP type, was mostly a poorly differentiated adenocarcinoma. Indeed, the IHC data on GATA6 (Classical marker) and CK5 (Basal-like marker) retained the properties of the “molecular” subtype of original tumors (Fig. 2C).

### RNA-seq analysis of PDOs

We conducted an RNA-seq analysis of PDAC PDOs. Each PDO reproduced the original sample in terms of transcriptome signatures (Supplementary Fig. S3). We confirmed that GL and DP could be separated by both principal component analysis and hierarchical clustering (Supplementary Fig. S4A). We identified 1711 differentially expressed genes (DEGs) between the GL and DP types. DEGs of GL type high included not only the representative “Classical” signatures, such as *GATA6*, *SPINK4*, *TFF1*, *AGR2*, and *PDX1*, but also the “Exocrine-like” (or “ADEX”) signatures, such as *REG3A*, *REG1A*, and *CFTR*. In contrast, DEGs of DP type high included “Basal-like” (or “Squamous”) signatures, such as *KRT5*, *CAV1*, *S100A2*, and *LEMD1*, and those related to WNT and transforming growth factor β (TGF-β) signaling, immune evasion (such as interleukin *[IL]34* and *IL20RB*), and angiogenesis (Fig. 2D).

GSEA showed that gene sets of Progenitor PDAC- and Classical-signatures were enriched in the GL type (Fig. 2E, F). Genes related to oxidative phosphorylation and cholesterol homeostasis were upregulated in the GL type. In contrast, in DP cells, genes related to epithelial–mesenchymal transition (EMT), angiogenesis, inflammatory pathway, and cell proliferation, such as KRAS signaling, were upregulated. GO analysis of the DEGs revealed that those enriched in the GL type were lipogenic, whereas genes related to cell adhesion and EMT were enriched in the DP type (Supplementary Fig. S4B). *Protocadherins* (*Pcdhs*) and *homeotic* (*Hox*) *genes* were upregulated in the DP type.

### Correlation of “morphological” classification with clinical outcomes

Some previous studies have suggested that the “Classical” responds better to FFX (24, 26). To explore whether “morphological” classification predicts clinical outcomes, such as OS and treatment response, we performed a prospective registry analysis of PDOs and examined the correlation between “morphological” classification and clinical information. Among the 35 established PDOs, 20 were GL type (57%) and 15 were DP type (43%) (Supplementary Fig S5A, B). Stage and CA19-9 were significantly different between the GL and DP types; the GL type was dominant in resectable cases (Stage I/II), while the DP type was dominant in the metastatic cases, and CA19-9 was higher in the DP type than in the GL type (median [U/mL]: 913 vs. 267, *P* < 0.05; Supplementary Table S4). Furthermore, the DP type was especially dominant in the liver biopsy samples and ascites (Supplementary Fig. S5C).

**Fig. 3:**
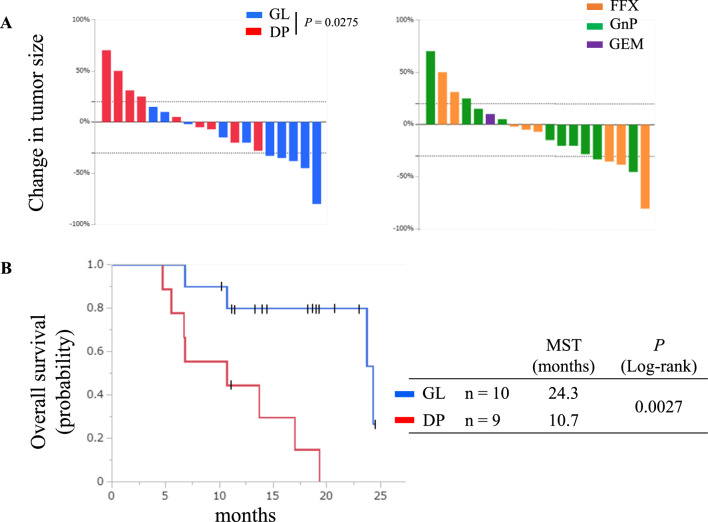
**A** Waterfall plots illustrating changes in tumor size on CT imaging (RECIST response) of unresectable cases receiving chemotherapy (*n* = 19) according to “morphological” classification (left) and chemotherapy regimen (right). *FFX* FOLFIRINOX, *GEM* gemcitabine, *GnP* GEM plus nab-paclitaxel (**B**), Kaplan–Meier OS curves of unresectable cases according to “morphological” classification. *MST* median survival time

During the follow-up of unresectable cases that received chemotherapy, there were ten GL and nine DP types. RECIST measurements were available 8–12 weeks after the initiation of chemotherapy. We found that the GL type was significantly correlated with a good response (*P* < 0.05, Fig. 3A). In particular, patients receiving FFX showed a high response rate. Conversely, no cases of the DP type reached even partial response (PR) in RECIST, and half did not achieve tumor shrinkage. Both GL and DP types showed no significant differences in background factors, including PS, stage, CA19-9, and tumor size (Supplementary Table S5); however, the median OS in patients with the GL type was significantly longer than that in patients with the DP type (24.3 vs. 10.7 months, *P* < 0.005, Fig. 3B, Supplementary Fig S5D). Thus, the “morphological” classification of PDOs was significantly correlated with the clinical treatment response and prognosis of the patients.

### Co-administration of ERK inhibitor and chloroquine

**Fig. 4:**
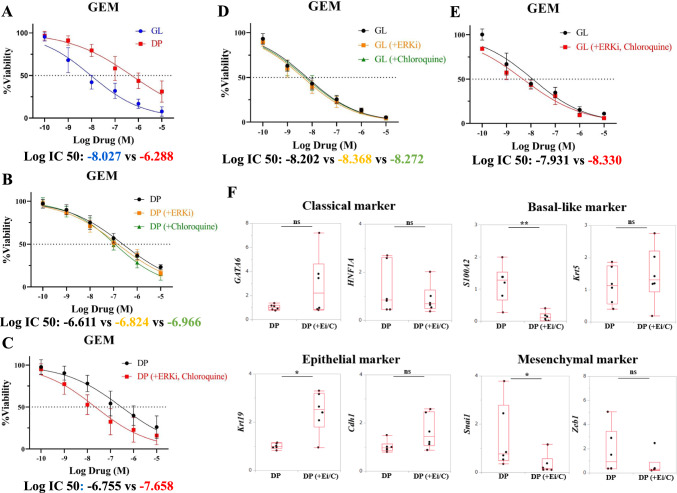
**A**–**E** Drug response assay of PDOs reveals heterogeneity of chemotherapy response. Dose–response curves for GEM according to GL vs. DP (**A**), ERK inhibition with GEM vs. chloroquine with GEM vs. only GEM in DP and GL (**B**, **D**), Co-administration of ERK inhibitor and chloroquine with GEM vs. only GEM in DP and GL (**C**, **E**). **F** Changes in gene expression to DP type after co-administration of ERK inhibitor and chloroquine. Box–whisker plots show median ± first and third quartiles. ns: not significant, *P < 0.05, **P < 0.01 + Ei/C: co-administration of ERK inhibitor and chloroquine

We performed a drug response assay using GEM as a key drug for PDAC treatment. We found that the response to GEM varied among PDOs obtained from different patients. The GL type showed a significantly better drug response than the DP type (Fig. 4A). We also explored drugs that can improve the treatment response of the DP type and found that treatment with GEM and either ERK or chloroquine slightly improved the IC_50_ of the DP type (log IC_50_: DP vs. DP + ERK inhibitor vs. DP + chloroquine, − 6.611 [− 6.765 to − 6.453] vs. − 6.824 [− 6.970 to − 6.677] vs. − 6.946 [− 7.102 to − 6.787]; Fig. 4B). Of note, the co-administration of ERK inhibitor and chloroquine markedly improved the response to GEM in the DP type. Co-administration improved the IC_50_ of DP type (log IC_50_: DP vs. DP + ERK inhibitor and chloroquine, − 6.526 [− 6.755 to − 6.286] vs. − 7.658 [− 7.887 to − 7.424]) and inhibited cell proliferation (Fig. 4C, Supplementary Fig. S5E). In addition, we observed a similar trend in the GL type, although the effect of co-administration was not as significant as in the DP type (Fig. 4D, E).

To confirm the changes in DP type with or without co-administration of ERK inhibitor and chloroquine, we examined the changes in gene expression using qPCR for representative subtype and EMT markers. We observed reduced expression of *S100A2* (Basal-like marker) and *Snai1* (mesenchymal marker) and increased expression of *Krt19* (epithelial marker; Fig. 4F).

## Discussion

PDAC is one of the most refractory cancers. Therefore, breakthroughs to improve patient outcomes are required. However, no appropriate animal models are available to recapitulate the diversity and disease processes of human PDAC for decades. In addition, as PDAC is rich in RNase, it is difficult to extract high-quality RNA from EUS-FNA samples [35]; hence, transcriptome analysis was mainly performed on surgical samples. PDOs have the potential to overcome these problems; by combining PDOs with omics analysis, remarkable progress has been made in the basic research of PDAC in recent years [19–22]. In this study, we successfully established PDOs of PDAC in a wide range of disease stages, from the early to advanced stages, using tumor samples obtained by EUS-FNB, liver biopsy, surgical resection, ERCP, and abdominal paracentesis. For clinical application, we comprehensively investigated the PDOs of PDAC from establishment to analysis through RNA-seq and drug response assays as well as a comparison of clinical information.

With the advances in cancer genome medicine, tumor samples obtained using EUS-FNB at the time of diagnosis have important clinical implications. However, to obtain sufficient amounts of tissue, multiple punctures should be made with a thicker size needle, which may result in serious complications such as bleeding [36, 37]. In addition, the current turnaround time in cancer genome medicine is too long for patients with PDAC. The establishment of PDOs in PDAC is difficult [38], and their application to clinical treatment is still in its infancy. In this study, we demonstrated that a cutoff of 100,000 live cells is important for establishing PDOs in PDAC and that EUS-FNB with a single puncture of the 22G needle is sufficient for establishing PDOs.

Our study revealed that the “morphological” classification of PDOs corresponds to the “molecular” subtypes. Regardless of genetic mutation, the gene expression pattern was correlated with “Classical” and “Exocrine-like” (or “ADEX”) in GL type and “Basal-like” and “Squamous” in DP type. The PDOs in our study reproduced the original samples in terms of transcriptome signatures, as reported previously [23, 39]. Tumor samples from EUS-FNB and liver biopsy reflect a part of original tumors and the possibility of selection pressure during culture cannot be completely eliminated; however, we consider that PDOs largely replicate the original tumors. Although the subtype model was reproduced via mouse transplantation in a previous report by Miyabayashi et al. [40], generalization is still difficult because of the problems of long reproduction time and the complexity of transplantation procedures. In this study, we propose a subtype model that can be reproduced more rapidly and easily by the morphological evaluation of PDOs. As a result, PDOs address another challenge in precision medicine: turnaround time. The subtypes can be determined in less than 2 weeks via morphologic evaluation of PDOs. Once established from a small number of tissue samples, PDOs can help in the selection of appropriate treatments in clinical practice.

We investigated the differences between the GL and DP types. In the GL type, the expression of genes involved in oxidative phosphorylation and cholesterol metabolism was increased, and we confirmed that the GL type maintained relatively differentiated pancreatic signatures. In contrast, in the DP type, the expression of “Squamous” markers such as *ZEB1*, *KRT5*, and *S100A2*, and genes related to EMT, WNT/TGF-β and KRAS signaling, angiogenesis, and cell proliferation were elevated. These observations suggest that the DP type is induced in the direction of EMT and has a highly malignant potential. These findings are consistent with previously reported subtype features [19, 23] and were reported to be poor prognostic factors for PDAC [41–43]. Moreover, we discovered additional significant features of the DP type, i.e., immune evasion and *Hox genes*. The expression of genes related to immune evasion, such as *IL-34* and *IL-20RB*, causes resistance to immune checkpoint blockade [44, 45], and increased expression of *IL20RB* is correlated with reduced survival in patients with PDAC [46]. Meanwhile, *Hox genes* contribute to cancer progression by sustaining proliferative signaling and tumor-promoting inflammation, inducing angiogenesis and resistance to chemotherapy, and preventing cell death [47, 48]. We consider these characteristics enhanced in the DP type, which is a “Basal-like” phenotype, to contribute to the intractability, treatment resistance, and poor prognosis of PDAC.

We compared “morphological” classification with clinical information to verify its usefulness as a subtype model. Previous reports showed that approximately 70% of the surgical samples were “Classical [15]” and that they shifted to “Basal-like” as the disease progressed [19, 21], while subtype-discordant tumors exhibit intermediate phenotypes [49]. Our cohort included a wide range of cases, from resectable to metastatic, resulting in an overall ratio of GL to DP of 4 to 3. Most resectable cases were GL, whereas half of the locally advanced cases and the majority of the metastatic cases were DP, especially in samples from liver metastases and ascites. These results are consistent with previous reports that PDAC progresses from “Classical” to “Basal-like” [19–21]. In terms of treatment response, RECIST PR cases were only GL, FFX showed especially good results, and all RECIST PD cases were DP. Furthermore, GL was associated with a significantly better prognosis than DP, even in patients with unresectable PDAC. Therefore, the selection of FFX for GL will likely result in better clinical outcomes. In particular, for resectable cases, in which GL accounts for the majority of cases, FFX might be useful as a neoadjuvant chemotherapy. Further analysis using real-world data is required to validate our results.

**Fig. 5:**
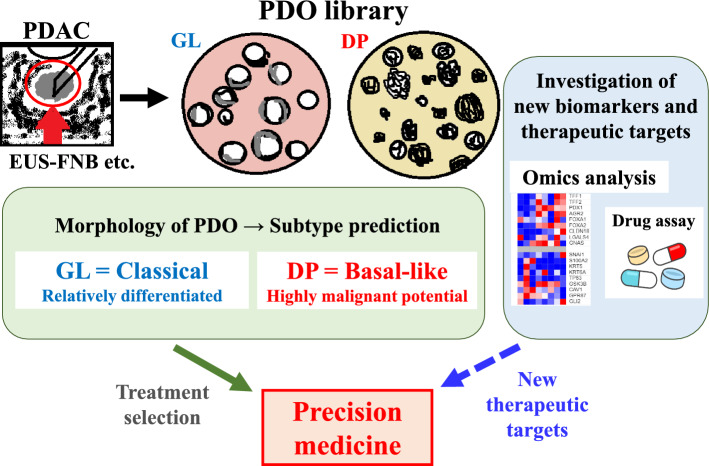
Graphical abstract depicting the model of precision medicine based on PDOs

Even in resected cases, the “Basal-like” subtype has a poor prognosis regardless of whether conventional pathological staging is favorable or not [50]. Thus, there is an urgent need to establish a therapeutic strategy for “Basal-like.” In this study, we discovered that as a novel therapeutic strategy for “Basal-like”, co-administration with both ERK inhibitor and chloroquine improved the response to GEM in PDOs of the DP type. In contrast, the GL type showed less benefit from co-administration. Chan-Seng-Yue et al. reported that downstream signaling of KRAS is enhanced as a result of amplification of KRAS mutation in “Basal-like” [19]. In PDAC with KRAS mutation, inhibition of the MAPK pathway promotes tumor-protective autophagy, and combination therapy with both ERK and autophagy inhibitors has a synergistic effect, whereas either alone has a weaker effect [51]. In our GSEA, KRAS signaling was enhanced in the DP type. Based on the results, we postulate that suppressing both MAPK signaling and autophagy can effectively suppress tumor growth in the “Basal-like” subtype. Interestingly, co-administration with both ERK inhibitor and chloroquine decreased the expression of “Basal-like” and mesenchymal markers, such as *S100A2* and *Snai1*, and increased the expression of *Krt19*, suggesting a transition toward the epithelial phenotype. Currently, there are no effective treatment strategies for “Basal-like.” This study suggests the potential of combination therapy in improving the malignant traits acquired by transitioning to “Basal-like” as the disease progresses. Further analyses using PDOs are required to validate the results of the present study. Figure 5 shows the graphic abstract of this study.

Despite the findings, this study has some limitations that should be considered. First, this was a single-center study, and the sample size was limited. Second, the observational period was too short to analyze long-term prognosis. Third, the whole genome (or exon) sequences from the enrolled patients were unavailable. Lastly, we did not address how chloroquine works in vitro and in vivo. Nonetheless, the concept of this study reduce turnaround time to determine subtype, facilitate the bench-to-bedside precision medicine, and might be lead to the development of new treatment strategy. We believe that the results of this study will be useful for clinical treatment of PDAC in the future.

In conclusion, EUS-FNB is useful in establishing PDAC PDOs. PDOs allow for subtype prediction based on morphological evaluation in the short term and can be used for optimal treatment selection. It is also possible to explore strategies to improve treatment for “Basal-like,” and new therapeutic targets such as combination therapy can be applied to achieve precision medicine in the long term.

### Supplementary Information

Below is the link to the electronic supplementary material.

**Supplementary Figure S1**. (A) Live cell counts obtained by each sampling method. (B) Comparing of using one puncture between EUS-FNB and liver biopsy. More live cells can be obtained using EUS-FNB than liver biopsy (right). No difference in tumor size was observed (left). EUS-FNB was useful for establishing PDOs. ns: not significant, **: P < 0.01 (C) Schema of each puncture needle. **Supplementary Figure S2**. (A) Major genetic mutations of PDAC inferred from RNA-seq. No significant differences were observed in the frequency between the two subtypes. (B) Gene expression levels around *SMAD4* and *CDKN2A*. Deletions of *SMAD4* and *CDKN2A* were detected in PDO47. (C) Minor genetic mutations in PDAC inferred from RNA-seq. **Supplementary Figure S3**. (A, B) Principal component analysis (PCA) and hierarchical clustering using the 200 most differentially expressed genes. Each PDO reproduced the original sample in terms of transcriptome signatures. (C) Heatmap of gene expression levels according to “Classical” and “Basal-like” signatures in original samples and paired PDOs. **Supplementary Figure S4**. (A) PCA (top) and hierarchical clustering using the 200 most differentially expressed genes (bottom). (B) GO analysis of DEGs (top). Representative findings for each group are presented. Hallmark pathways significantly enriched among DEGs in GL and DP according to GSEA (bottom). Gene sets with high NES are shown. **Supplementary Figure S5**. (A) Pie charts showing the proportions of GL and DP. (B) Representative images of GL and DP type. Scale bar, 100 µm. (C) For Stage IV cases, GL and DP proportions differed between sampling lesions. GL was dominant in primary lesions, while DP was particularly dominant in liver metastases and ascites. (D) Morphological evaluation of PDOs was positively correlated with OS. (E), Representative images of PDO treated by co-administration of ERK inhibitor and chloroquine with GEM (bottom), only GEM (middle), and control (top). Scale bar, 100 µm. (PPTX 2929 KB)

Supplementary file2 (DOCX 46 KB)

## Abbreviations

ACTB: Actin beta
AGR2: Anterior gradient 2
BRCA: Breast cancer gene
CAV1: Caveolin 1
CDH1: Cadherin 1
CFTR: Cystic fibrosis transmembrane conductance regulator
CK5: Cytokeratin 5
GATA6: GATA-binding protein 6
HNF1A: Hepatocyte nuclear factor 1 homeobox A
KRAS: V-Ki-ras2 Kirsten rat sarcoma viral oncogene homolog
KRT5, 19: Keratin 5, 19
LEMD1: LEM domain-containing 1
PDX1: Pancreatic–duodenal homeobox factor-1
REG4, 3A, 1A: Regenerating islet-derived family member 4, 3A, 1A
SNAI1: Snail family transcriptional repressor 1
SPINK4: Serine peptidase inhibitor kazal type 4
S100A2: S100 calcium-binding protein A2
TFF1: Trefoil factor family 1
ZEB1: Zinc finger E-box-binding homeobox 1
