# Supplementary material for: Patient-derived organoids of pancreatic ductal adenocarcinoma for subtype determination and clinical outcome prediction
Source: J Gastroenterol. 2024 Apr 29;59(7):629–40. doi: 10.1007/s00535-024-02103-0 (PMC11217054; doi:10.1007/s00535-024-02103-0)
Supplement: Supplementary file 1 — Supplementary Figure S1. (A) Live cell counts obtained by each sampling method. (B) Comparing of using one puncture between EUS-FNB and liver biopsy. More live cells can be obtained using EUS-FNB than liver biopsy (right). No difference in tumor size was observed (left). EUS-FNB was useful for establishing PDOs. ns: not significant, **: P < 0.01 (C) Schema of each puncture needle. Supplementary Figure S2. (A) Major genetic mutations of PDAC inferred from RNA-seq. No significant differences were observed in the frequency between the two subtypes. (B) Gene expression levels around SMAD4 and CDKN2A. Deletions of SMAD4 and CDKN2A were detected in PDO47. (C) Minor genetic mutations in PDAC inferred from RNA-seq. Supplementary Figure S3. (A, B) Principal component analysis (PCA) and hierarchical clustering using the 200 most differentially expressed genes. Each PDO reproduced the original sample in terms of transcriptome signatures. (C) Heatmap of gene expression levels according to “Classical” and “Basal-like” signatures in original samples and paired PDOs. Supplementary Figure S4. (A) PCA (top) and hierarchical clustering using the 200 most differentially expressed genes (bottom). (B) GO analysis of DEGs (top). Representative findings for each group are presented. Hallmark pathways significantly enriched among DEGs in GL and DP according to GSEA (bottom). Gene sets with high NES are shown. Supplementary Figure S5. (A) Pie charts showing the proportions of GL and DP. (B) Representative images of GL and DP type. Scale bar, 100 µm. (C) For Stage IV cases, GL and DP proportions differed between sampling lesions. GL was dominant in primary lesions, while DP was particularly dominant in liver metastases and ascites. (D) Morphological evaluation of PDOs was positively correlated with OS. (E), Representative images of PDO treated by co-administration of ERK inhibitor and chloroquine with GEM (bottom), only GEM (middle), and control (top). Scale bar, 100 µm. (PPTX 2929 KB) [file 535_2024_2103_MOESM1_ESM.pptx]

## Slide 1
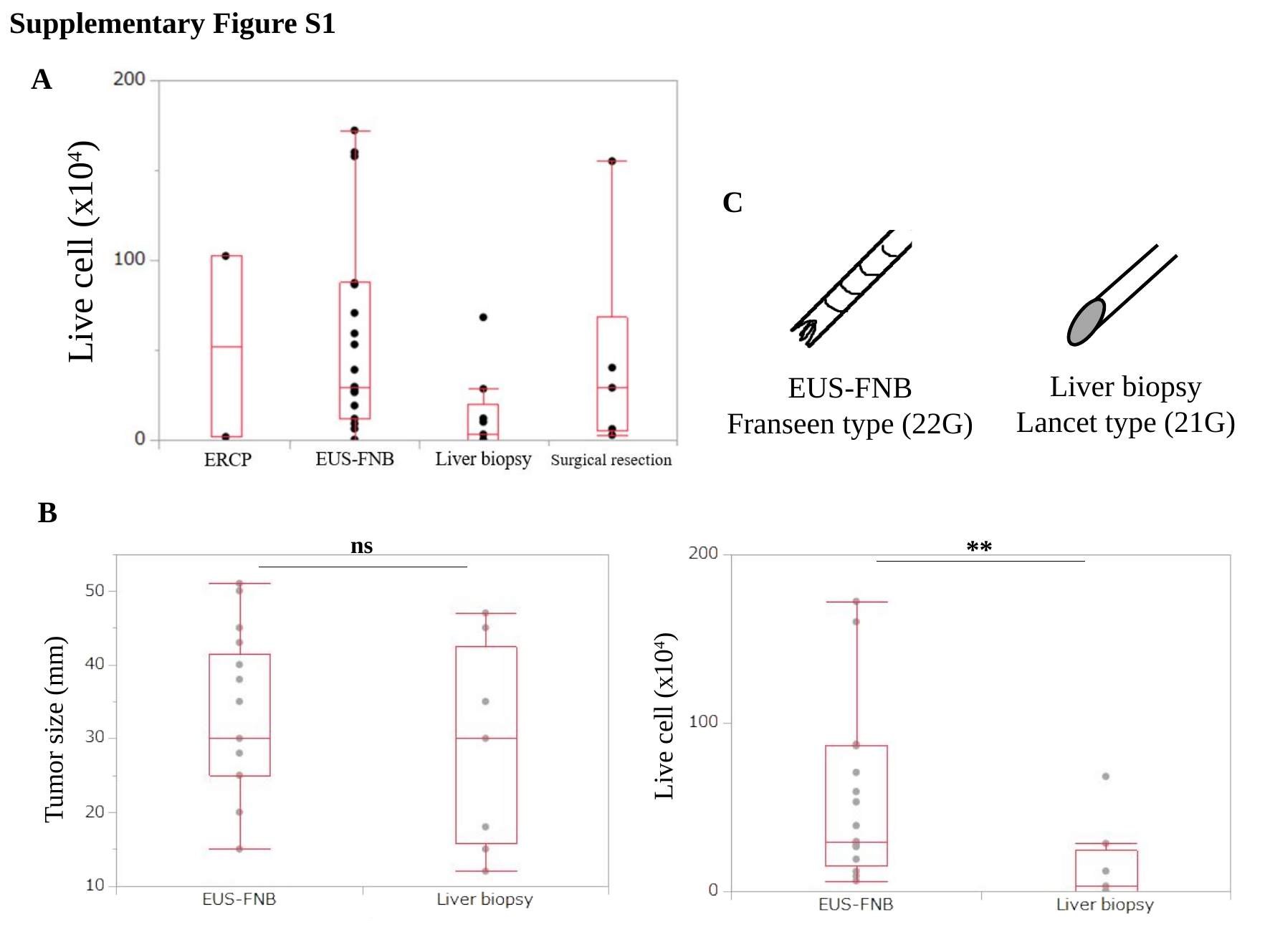

Supplementary Figure S1
A
Live cell (x104)
C
Liver biopsy
Lancet type (21G)
EUS-FNB
Franseen type (22G)
B
ns
**
Live cell (x104)
Tumor size (mm)

## Slide 2
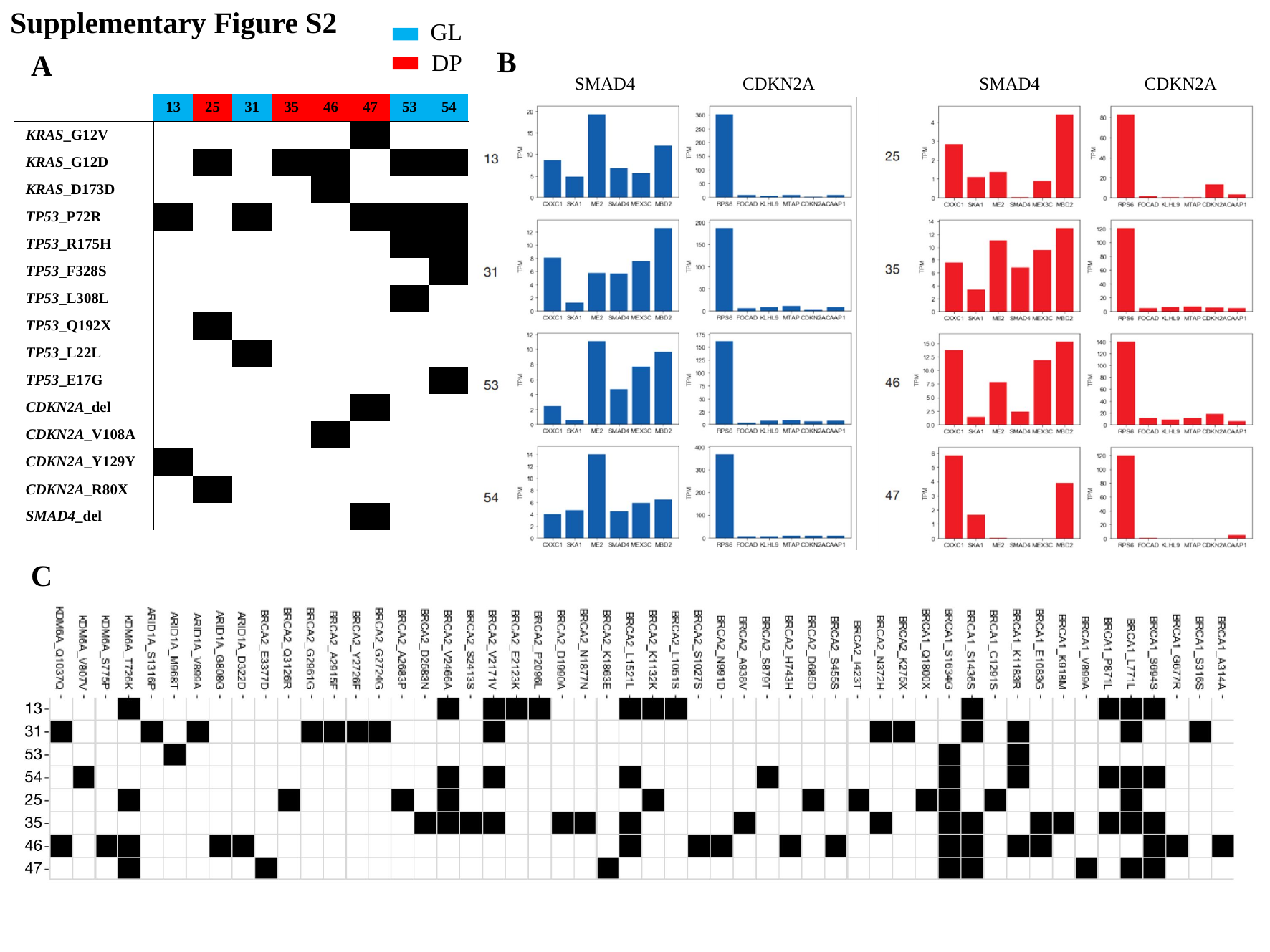

Supplementary Figure S2
GL
B
A
DP
SMAD4
CDKN2A
SMAD4
CDKN2A
| | 13 | 25 | 31 | 35 | 46 | 47 | 53 | 54 |
| --- | --- | --- | --- | --- | --- | --- | --- | --- |
| KRAS\_G12V | | | | | | | | |
| KRAS\_G12D | | | | | | | | |
| KRAS\_D173D | | | | | | | | |
| TP53\_P72R | | | | | | | | |
| TP53\_R175H | | | | | | | | |
| TP53\_F328S | | | | | | | | |
| TP53\_L308L | | | | | | | | |
| TP53\_Q192X | | | | | | | | |
| TP53\_L22L | | | | | | | | |
| TP53\_E17G | | | | | | | | |
| CDKN2A\_del | | | | | | | | |
| CDKN2A\_V108A | | | | | | | | |
| CDKN2A\_Y129Y | | | | | | | | |
| CDKN2A\_R80X | | | | | | | | |
| SMAD4\_del | | | | | | | | |
C

## Slide 3
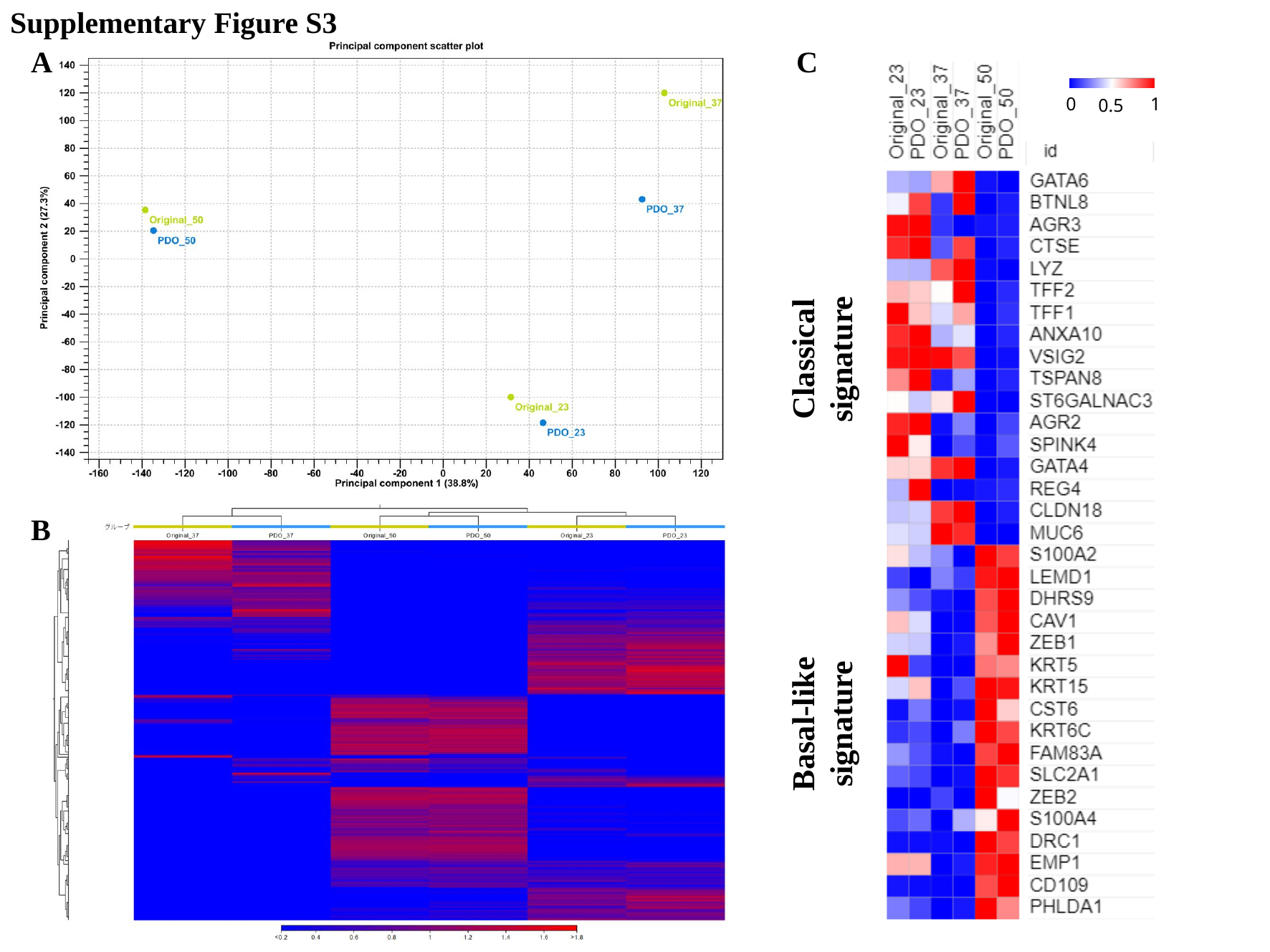

Supplementary Figure S3
A
C
1
0
0.5
Classical
signature
B
Basal-like
signature

## Slide 4
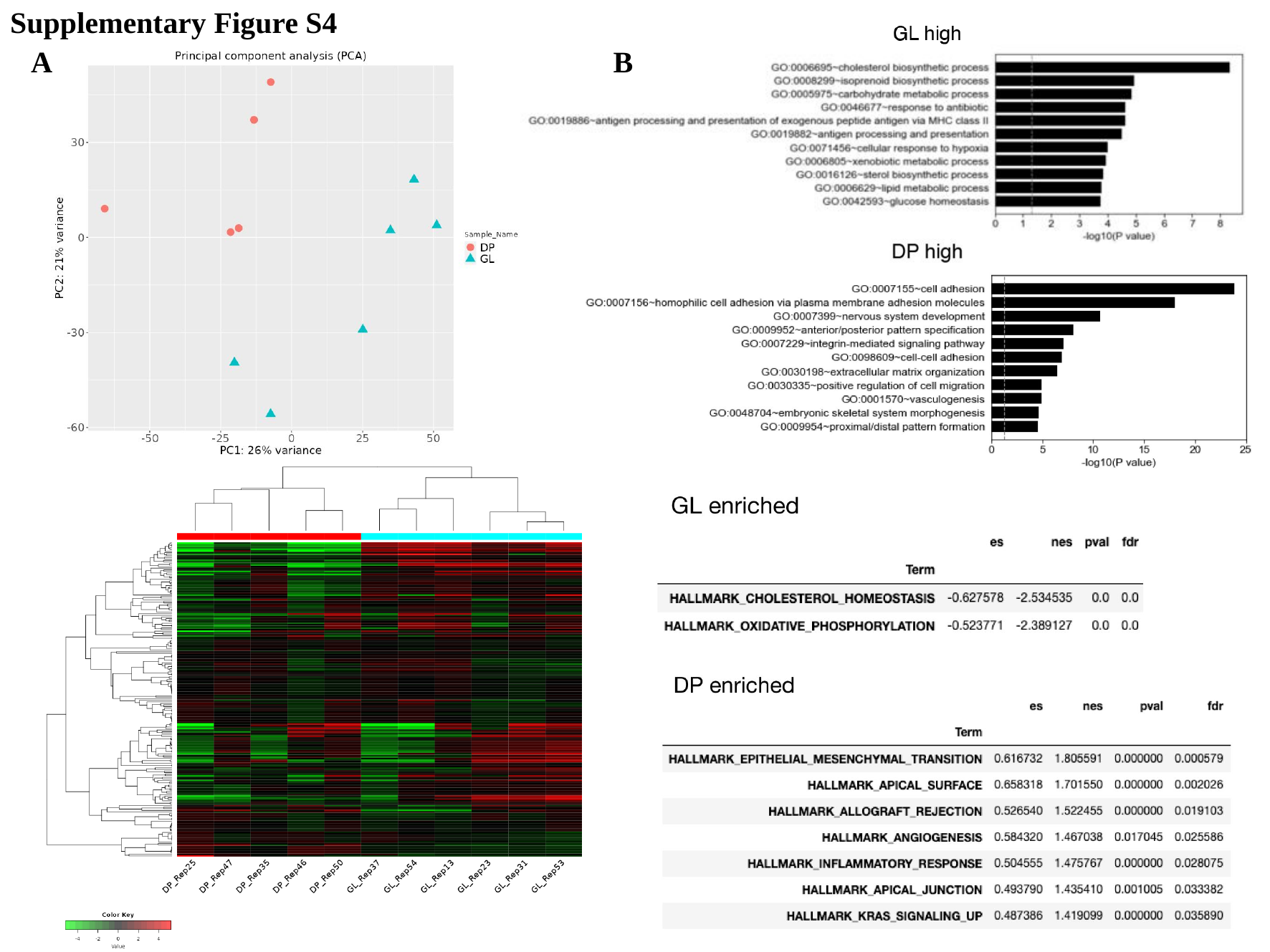

Supplementary Figure S4
A
B

## Slide 5
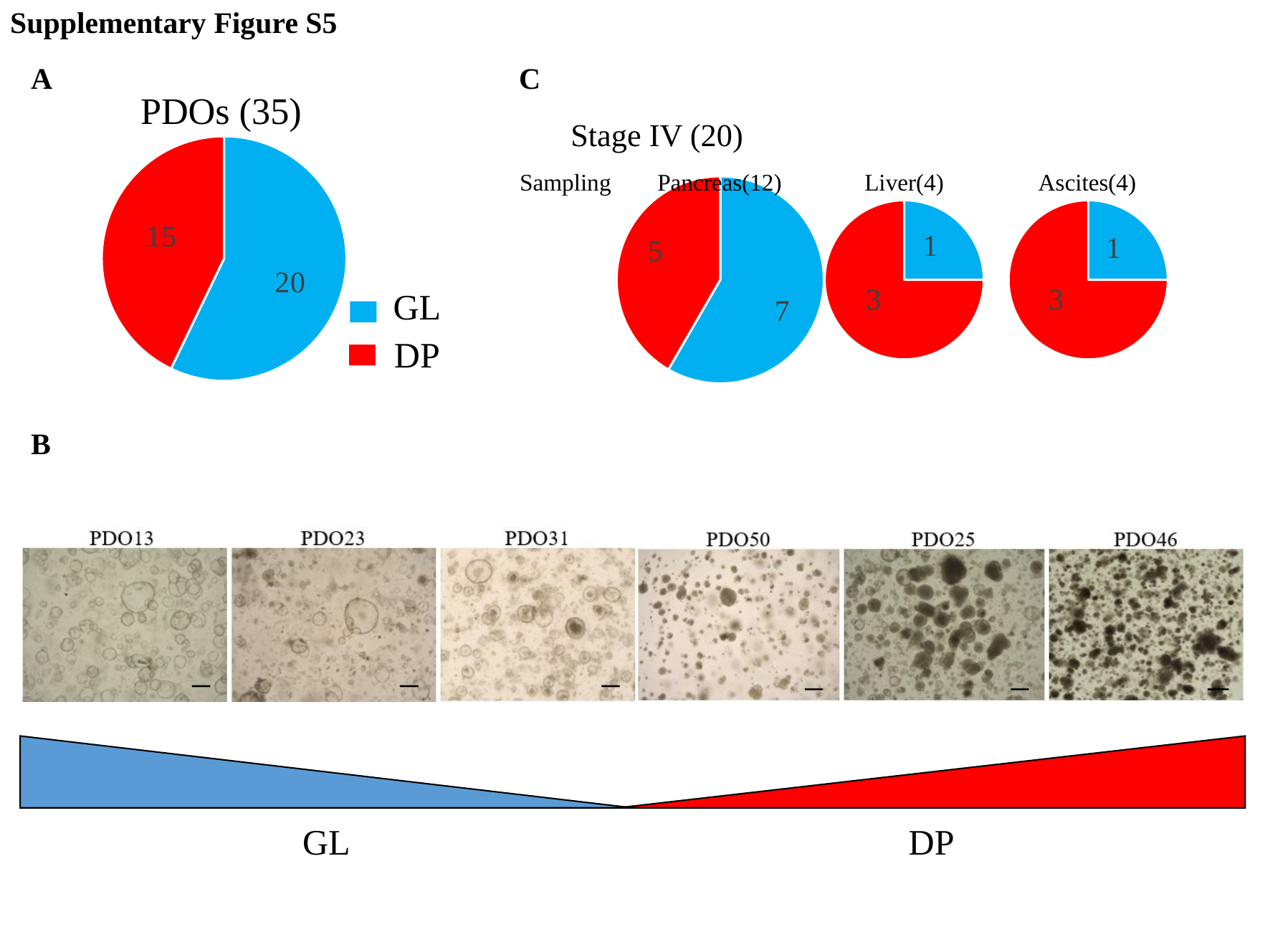

Supplementary Figure S5
A
C
PDOs (35)
Stage IV (20)
### Chart
| Category | 列1 |
|---|---|
| | 20.0 |
| | 15.0 |Sampling
Pancreas(12)
Liver(4)
Ascites(4)
### Chart
| Category | 列1 |
|---|---|
| | 7.0 |
| | 5.0 |
### Chart
| Category | 列1 |
|---|---|
| | 1.0 |
| | 3.0 |
### Chart
| Category | 列1 |
|---|---|
| | 1.0 |
| | 3.0 |GL
DP
B
GL
DP

## Slide 6
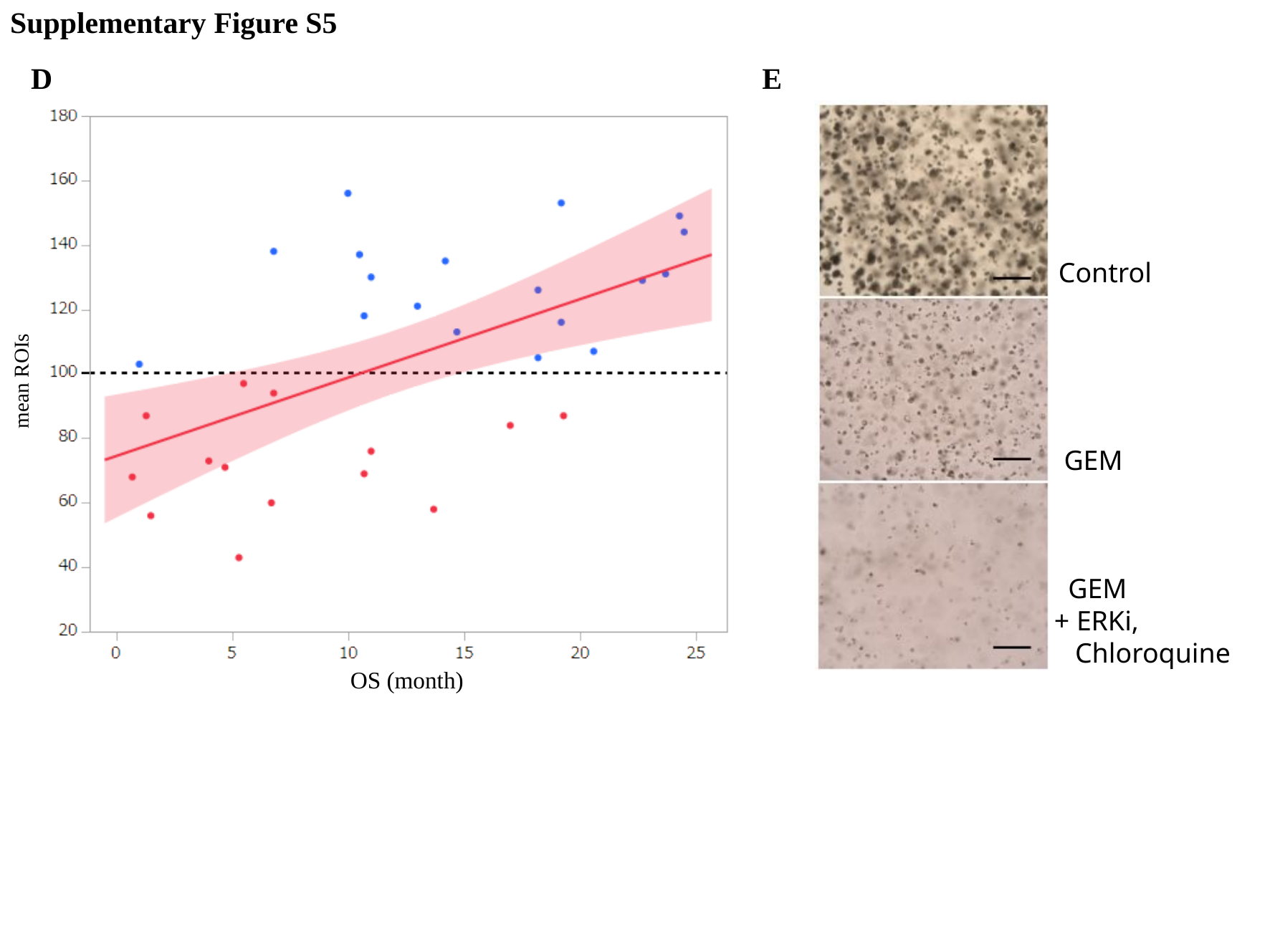

Supplementary Figure S5
D
E
Control
mean ROIs
GEM
 GEM
+ ERKi,
 Chloroquine
OS (month)
GL
DP
