# Supplementary material for: Patient-derived organoids of pancreatic ductal adenocarcinoma for subtype determination and clinical outcome prediction
Source: J Gastroenterol. 2024 Apr 29;59(7):629–40. doi: 10.1007/s00535-024-02103-0 (PMC11217054; doi:10.1007/s00535-024-02103-0)
Supplement: Supplementary file 2 — Supplementary file2 (DOCX 46 KB) [file 535_2024_2103_MOESM2_ESM.docx]

**Supplementary Table S1: Reagents and resources**

| **<Basal medium>** |  | Source | Identifier |
| --- | --- | --- | --- |
| Advanced DMEM/F12 | 500 mL | Thermo Fisher Scientific | 12634010 |
| HEPES | 5 mL | Thermo Fisher Scientific | 15630080 |
| Penicillin- Streptomycin Mixed Solution | 5 mL | Nacalai | 09367-34 |
| GlutaMAX^TM^ (100X) | 5 mL | Thermo Fisher Scientific | 35050061 |
| **<Complete medium>** |  |  |  |
| Basal medium | 8 mL |  |  |
| Afamin-Wnt3A CM | 1 mL | MBL | J2-001 |
| R-spondin-1 CM | 1 mL | RSD | 3710-001-01 |
| EGF | 500 ng | Thermo Fisher Scientific | PMG8041 |
| Noggin | 1 µg | Pepro Tech | 250-38 |
| IGF-1 | 1 µg | Bio Legend | 590904 |
| FGF-2 | 500 ng | Pepro Tech | 100-18B |
| A83-01 | 500 nM | R&D Systems | 2939/10 |
| Y-27632 | 10 µM | FUJIFILM WAKO | 25900613 |
| B-27 supplement | 200 µL | Thermo Fisher Scientific | 17504044 |
| N-acetyl-L-cysteine | 1 mM | Sigma-Aldrich | A9165 |
| [Leu^15^] Gastrin I | 10 nM | Merck | G9145 |
| **<Others>** |  |  |  |
| Liberase TH Research Grade |  | Roche | 5401135001 |
| TrypLE Express |  | Thermo Fisher Scientific | 12605010 |
| RBC Lysis Buffer |  | Roche | 11814389001 |
| Recovery Cell Culture  -Freezing Medium |  | Thermo Fisher Scientific | 12648010 |

**Supplementary Table S2: List of primers used in RT-PCR**

| Primers | Sequence (5' to 3') |
| --- | --- |
| *ACTB* (F) | CCAACCGCGAGAAGATGA |
| *ACTB* (R) | CCAGAGGCGTACAGGGATAG |
| *GATA6* (F) | GCGAGCGCTGTTTGTTTAG |
| *GATA6* (R) | TTACTGCTCTGCCGGAAAAC |
| *HNF1A* (F) | ACGACGATGGGGAAGACTTC |
| *HNF1A* (R) | GACTTGACCATCTTCGCCAC |
| *S100A2* (F) | GAACTTCTGCACAAGGAGCTG |
| *S100A2* (R) | AAAGGCATCAACAGTCCTGGG |
| *KRT5* (F) | TCCAGTGTGTCCTTCCGAAGT |
| *KRT5* (R) | TGCCTCCGCCAGAACTGTA |
| *KRT19* (F) | TTTGAGACGGAACAGGCTCT |
| *KRT19* (R) | AGCTCTTCCTTCAGGCCTTC |
| *CDH1* (F) | GACTCGTAACGACGTTGCAC |
| *CDH1* (R) | GCCGCTTTCAGATTTTCATC |
| *SNAI1* (F) | ACCCACACTGGCGAGAAG |
| *SNAI1* (R) | TGACATCTGAGTGGGTCTGG |
| *ZEB1* (F) | TGTGGTAGAAACAAATTCAGATTC |
| *ZEB1* (R) | GCCCTTCCTTTCCTGTGTCA |

**Supplementary Table S3: Patient characteristics for RNA-seq analysis**

| PDO | Age(y) / Sex | CA19-9  (U/mL) | Size  (mm) | Sampling | Stage,  UICC 7^th^ | Metastasis | Treatment | REC-IST | OS (M) |
| --- | --- | --- | --- | --- | --- | --- | --- | --- | --- |
| 13  (GL) | 65 / M | 3221 | 20 | EUS-FNB | IV | Liver | mFFX | PR | 24 |
| 23  (GL) | 80 / F | 43.5 | 20 | Surgical  resection | IIA | - | NAC(GS)  ⇒ Ope | SD | 23  (Alive) |
| 31  (GL) | 77 / F | 133 | 35 | EUS-FNB | IIA | - | NAC(GnP)  ⇒ Ope | PR | 19  (Alive) |
| 37  (GL) | 77 / F | 66.1 | 25 | Surgical  resection | IIA | - | Ope | - | 19  (Alive) |
| 53  (GL) | 76 / M | 5 | 30 | EUS-FNB | IIA | - | NAC (GS)  ⇒ Ope | SD | 11  (Alive) |
| 54  (GL) | 74 / M | <2 | 5 | ERCP | I | - | Ope | - | 10  (Alive) |
| 25  (DP) | 66 / M | 4291 | 30 | Liver biopsy | IV | Liver | nal-ILI+  5-FU | PD | 5 |
| 35  (DP) | 71 / F | 7080 | 23 | EUS-FNB | IV | Liver, Lung, Bone | GnP | SD | 6 |
| 46  (DP) | 64 / F | 2020 | 51 | EUS-FNB | III | - | GnP | PD | 7 |
| 47  (DP) | 76 / F | 1550 | - | Ascites | IV | Peritoneal Dissemina-tion | BSC | - | 2 |
| 50  (DP) | 56 / M | 73.8 | 12 | Surgical  resection | IIA | - | NAC (mFFX)  ⇒ Ope | SD | 4 |

**Supplementary Table S4: Patient characteristics of GL and DP**

|  | PDOs (n＝35) | | *P* |
| --- | --- | --- | --- |
|  | GL (n=20) | DP (n=15) |  |
| Gender,  male / female | 12 / 8 | 9 / 6 | 1 |
| Age (median) | 67 (46- 81) | 65 (46- 76) | 0.4621 |
| Stage  (UICC 7th) |  |  | 0.048* |
| I / II | 8 | 1 |  |
| III | 3 | 3 |  |
| IV | 9 | 11 |  |
| CA19-9  (median, U/mL) | 267  (0- 3221) | 913  (30.7- 7080) | 0.0314* |

**Supplementary Table S5: Characteristics** **of patients with unresectable PDAC receiving chemotherapy**

|  | PDOs (n＝19) | | *P* |
| --- | --- | --- | --- |
|  | GL (n=10) | DP (n=9) |  |
| Gender,  male / female | 5 / 5 | 5 / 4 | 1 |
| Age (median) | 64 (46- 81) | 69 (56- 74) | 0.7743 |
| Stage  (UICC 7th) |  |  | 1 |
| III | 3 | 2 |  |
| IV | 7 | 7 |  |
| CA19-9  (median, U/mL) | 518  (0- 3221) | 435  (30.7- 5310) | 0.9024 |
| Sampling method |  |  | 0.582 |
| EUS-FNB | 9 | 7 |  |
| Liver biopsy | 1 | 2 |  |
| Tumor size  (median, mm) | 35 (20- 50) | 30 (15- 51) | 0.6518 |
